# Systems Pharmacology Based Study of the Molecular Mechanism of SiNiSan Formula for Application in Nervous and Mental Diseases

**DOI:** 10.1155/2016/9146378

**Published:** 2016-12-12

**Authors:** Xia Shen, Zhenyu Zhao, Xuan Luo, Hao Wang, Benxiang Hu, Zihu Guo

**Affiliations:** ^1^College of Pharmacy, Shaanxi University of Chinese Medicine, Xi'an, Shaanxi 712046, China; ^2^Bioinformatics Center, College of Life Science, Northwest A&F University, Yangling, Shaanxi 712100, China

## Abstract

*Background.* Mental disorder is a group of systemic diseases characterized by a variety of physical and mental discomfort, which has become the rising threat to human life. Herbal medicines were used to treat mental disorders for thousand years in China in which the molecular mechanism is not yet clear.* Objective.* To systematically explain the mechanisms of SiNiSan (SNS) formula on the treatment of mental disorders.* Method.* A systems pharmacology method, with ADME screening, targets prediction, and DAVID enrichment analysis, was employed as the principal approach in our study.* Results.* 60 active ingredients of SNS formula and 187 mental disorders related targets were discovered to have interactions with them. Furthermore, the enrichment analysis of drug-target network showed that SNS probably acts through “multi-ingredient, multitarget, and multisystems” holistic coordination in different organs pattern by indirectly regulating the nutritional and metabolic pathway even their serial complications.* Conclusions.* Our research provides a reference for the molecular mechanism of medicinal herbs in the treatment of mental disease on a systematic level. Hopefully, it will also provide a theoretical basis for the discovery of lead compounds of natural medicines for other diseases based on traditional medicine.

## 1. Introduction

According to WHO survey, “mental disorders” is a kind of complex disease combined with many different single diseases, which included depression, schizophrenia, autism dementia, and other psychoses. Meanwhile, 350 million people are affected by depression and 60 million people are affected by the bipolar affective disorder. The morbidity of autism, dementia, schizophrenia, and other psychoses is still staying at a fairly high rate in recent years (http://www.who.int/mediacentre/factsheets/fs396/en/) [1], which reduced the people's health level and living standard.

At present, selective serotonin reuptake inhibitor (SSRI) and selective serotonin-norepinephrine reuptake inhibitors (SNRIs) are widely used for treating many psychiatric symptoms and mental disorders, such as fluoxetine [2], duloxetine, and paroxetine [3]. These drugs can selectively inhibit the uptake of serotonin (5-HT) or norepinephrine (NE) from the synaptic gap. They have the function of relieving depressive symptoms by increasing the neurotransmitters. For example, clozapine has been widely utilized for schizophrenia treatment, and efficacy trials have demonstrated the reductive effect in psychotic symptoms [4]. Bumetanide is an adjuvant drug for treating children autism [5]. However, these medicines still have lots of significant toxic and side effects, such as nausea, vomiting, mydriasis, hypersalivation, and hyperthermia [6]. Improper use of clozapine may lead to various side effects, agranulocytosis, myocarditis, cardiomyopathy, lowered seizure threshold, and metabolic syndrome. Thus, their side effects often limit their use and may increase the mortality [7]. Meanwhile, these SSRI and SNRI did not show the significant therapeutic effect on the complications of mental disorders. Therefore, we urgently need to find a solution for the treatment of mental disorder and its complications.

SNS is one of basic formulas from* Shang-Han-Lun* written by Zhang Zhongjing, which contains four herbal medicines (*Radix Bupleuri*,* Radix Paeoniae Alba*,* Immature Bitter Orange*, and* Radix Glycyrrhizae*). Previous literature study showed that SNS exert salutary effects on chronic restraint stress related disorders [8] and hepatic protection [9]. But the active ingredients and the holistic treatment mechanism of SNS formula applied in mental disorders remain unclear.

The molecular treatment mechanism of TCM often set up a barrier with a complex and systems characteristic for modern pharmacology study. However, systems pharmacology method is an effective approach that can help researchers to understand the microcosmic mechanism of these four herbal medicines. It has been used in clarifying numerous TCM formulas in many complex diseases, such as Radix Curcuma formula [10] Xiao-Chai-hu-Decoction and Da-Chai-hu-Decoction [11] and cardiovascular diseases [12]. Thus, the application of systems pharmacology in our study may provide new opportunity to understand the TCM holistic treatment mechanism in series similar mental disorders. And it also provide a new way to discover the prodrug from TCM.

In our present work, a systems pharmacology method, combined with oral bioavailability (OB) prediction, drug-likeness (DL) evaluation, and blood-brain barrier (BBB) prediction, was employed to predict the active ingredients from SNS formula. In addition, the potential biological targets of these active ingredients and the interaction network were constructed by a novel weighted ensemble similarity (WES) algorithm. This algorithm has been used in many researches in TCM, such as Xijiao Dihuang decoction [13] and Danggui-shaoyao san decoction [14]. Finally, through analyzing the network constructed by Cytoscape 3.1.1, DAVID enrichment analysis and literature research, we preliminarily deciphered the holistic treatment mechanism of SNS formula in mental disorders and its complications.

## 2. Materials and Methods

### 2.1. Materials

The ingredients structures of four herbal medicines in SNS formula were collected from Traditional Chinese Medicines for Systems Pharmacology Database and Analysis Platform (TCMSP http://lsp.nwsuaf.edu.cn/tcmsp.php), NCBI PubChem database (https://pubchem.ncbi.nlm.nih.gov/), and wide-scale literature mining.

### 2.2. Active Ingredients Prediction

As is known to all, numerous medical plants constitute the formula of TCM. But it is not every natural product in the medicinal plant has a pharmacological activity. In order to explain the potential active natural products from TCM formula, the previous prediction is very necessary for the drug development process. And accurate identification of the active ingredients from herbal medicines is a fundamental step to assess the therapeutic mechanism of TCM formula. Therefore, the pharmacokinetic characteristics research of SNS formula ingredients would help us to understand the molecular mechanisms of them. Thus, we use the ADME screening in this step. This method mainly contains 3 parts in our research. It is a series of pharmacokinetic parameters calculated to describe the ingredients from four herbal medicines including oral bioavailability (OB), drug-likeness (DL), and blood-brain barrier (BBB) value, which is shown as follows.

#### 2.2.1. Oral Bioavailability Calculation

Oral bioavailability is usually used to determine the orally administered drugs which could overcome several barriers and delivery into systemic circulation. A computer model OBioavail which integrates with the metabolism (Cyptochrome P450 3A4, 2D6) and transport (P-glycoprotein) information to predict the OB value of herbal ingredient [15]. In our present work, we chose the ingredients with OB ≥ 30% as the candidate molecules for next study. The threshold is used here mainly in order to (1) screen potential oral absorption and utilization of ingredients from SNS formula and (2) simplify the ingredients for the next step to assess the holistic therapeutic mechanism from four herbal medicines.

#### 2.2.2. Drug-Likeness Calculation

Drug-likeness (DL) prediction can help us to remove the ingredients deemed to be chemically unsuitable for drug here. It can be deduced that it affects the absorption, distribution, metabolism, and excretion of the herbal medicine ingredients in the human body. This model was based on Tanimoto similarity defined as follows:

(1) $$\begin{array}{c} T\left(\;A,B\;\right)=\frac{A\cdot B}{{\left|A\right|}^{2}+{\left|B\right|}^{2}-A\cdot B}. \end{array}$$

In the formula above, “*A*” represents the ingredients from four herbal medicines, and “*B*” represents the average drug-likeness index of all 6511 molecules in DrugBank database based on Dragon software descriptors. The DL value represents the possibility of the compound which may possess certain biological properties. In our work, we choose the ingredients with suitable DL (DL ≥ 0.18), because the average DL index of DrugBank molecules is 0.18.

#### 2.2.3. Blood-Brain Barrier Penetrability Prediction

Considering the blood-brain barrier (BBB) is perhaps the most selective and tight barrier in the whole body, reflecting the brain's critical roles in regulating metabolism and cognition, and coordinating the functions of organs [16], whether a drug can regulate cognition or mental disorders, it is needed to consider whether the compound is able to penetrate the blood-brain barrier. Thus, a reliable BBB model was employed to examine whether the ingredients of four herbal medicines pass through the BBB. In this model, 190 related molecules which are either penetrating or nonpenetrating pass the BBB which was used to constitute the dataset. Next, we divided the ingredients from four herbal medicines into two parts. One of them with value more than −0.3 was BBB considered as penetrating and the other part with value less than −0.3 was considered as BBB nonpenetrating.

### 2.3. Potential Treatment Targets Prediction

In order to identify the molecular targets, a novel weighted ensemble similarity (WES) algorithm was employed to predict the potential treatment targets of 60 potential ingredients [17]. This model was built on a large dataset involving 98,327 drug-target relations based on BindingDB (http://www.bindingdb.org/bind/index.jsp), DrugBank (https://www.drugbank.ca/), PDB database (http://www.rcsb.org/pdb/), and GoPubMed (https://www.ncbi.nlm.nih.gov/). And the algorithm mainly contains three parts. (1) Identify the key ligand structural and physicochemical features by CDK and Dragon software. (2) In order to improve the accuracy, the overall similarities were converted into the size-bias-free normalized values to eliminate the relevant similarities from random. (3) Finally, Bayesian network was used to predict the ensemble similarities (*Z* score). Then we chose the targets, which score greater than 5, as the potential targets.

Next, the BLAST tool in UniProt database (http://www.uniprot.org/blast/) was employed to standardize the target related genes and screen the targets from the human. Then the target information was delivered to DAVID (https://david.ncifcrf.gov/), to locate the main expressive organ of these targets.

### 2.4. Molecule Mechanism Network Construction

At first, the related disease information based on the targets above can be obtained from PharmGKB (https://www.pharmgkb.org/index.jsp), CTD database (http://ctdbase.org/), and TTD database (http://bidd.nus.edu.sg/group/cjttd/). All of the relationships between ingredients-targets and targets-diseases constitute the Compound-Target (C-T) network and Target-Disease (T-D) network. Then the networks were generated by Cytoscape 3.3.0. It is a powerful bioinformatics software for data visualization and analysis. The network topological properties of the networks are analyzed by the plug-in of Cytoscape software.

Secondly, the related disease information of the targets was classified from Medical Subject Headings (MeSH) (https://www.nlm.nih.gov/mesh/MBrowser.html). After that, we integrated the disease information obtained from the MeSH to the T-D network.

## 3. Results and Discussion

### 3.1. Active Ingredients

We collected totally 734 compounds of four herbal medicines in SNS formula. After the ADME screening, we identified 60 potential active ingredients (OB ≥ 30, DL ≥ 0.18) including 9 from* Radix Bupleuri, *34 from* Radix Glycyrrhizae*, 8 from* Radix Paeoniae Alba*, and 9 from* Immature Bitter Orange*. The name, CAS code, and calculation parameter of these ingredients were shown in Table S1 in Supplementary Material available online at http://dx.doi.org/10.1155/2016/9146378.

As for mental disorders, brain and nervous system often plays the important role in the occurrence of it, such as anxiety, depression, schizophrenia, epilepsy, autism spectrum disorder, and attention deficit hyperactivity disorder [1]. Therefore, we consider that treatment effect of the herbal medicine ingredients may be through two aspects. Some of the ingredients mainly aimed at the brain, while another part act on the liver and other organs. Meanwhile, TCM formula often emphasizes compatibility of medicine and comprises multiple herbs rather than single herbal medicine because the synergistic effects of these herbal medicines are greater than the sum of the individual effects [18]. Therefore, discovering of the herbal medicine ingredients which can play the synergistic reaction in different organs becomes the first step of our study. We use the mathematical model to screen the potential active ingredients and distinguish the compounds into BBB penetrating and BBB nonpenetrating groups. The active ingredients would be made clear in two parts of our work. There are 34 ingredients (BBB ≥ −0.3) which are performed BBB penetrating and the other 26 of them (BBB ≤ −0.3) are performed BBB nonpenetrating. Meanwhile, the ingredients which were divided into two parts may have different functions. According to our literature research, we found it mainly through two aspects. One is that the ingredients with BBB penetrating performed the curative effect for the main disease and symptoms. And the other ingredients with BBB nonpenetrating have the therapeutic effect not only on the primary disease, but also on the complications, which are specified as follows.

*Radix Bupleuri* as the main herbal medicine of SNS formula plays the most important role in the therapeutical effect of mental disorders. In order to prove it, we found that some of the ingredients have been reported as active ingredients, such as stigmasterol (CH8, 43.83%, 0.76) and cubebin (CH60, 57.13%, 0.64) which exhibit the antidepressant-like effect [19]. Stigmasterol was reported to have the function of slowing sensory nerve and nervous conduction velocity as well as CNS depression [20]. Previously, cubebin was reported to have the function of anti-inflammatory effect [21], and anti-inflammatory drugs also have potential antidepressant activity [22]. Since it has not been reported to have the direct antidepressant effect, therefore, we consider cubebin, as a component of* Radix Bupleuri* to have the effect of antidepressant as an active ingredient. In addition, saikosaponins, the other kinds of active ingredients from* Radix Bupleuri*, had already been experimented that it can augment the diminished monoamine neurotransmitter in the prefrontal cortex induced by chronic mild stress (CMS) [23] and have an antidepressant effect [24]. Thus, Saikogenin G (CH29, 51.84%, 0.63) and the hydrolysate of saikosaponins (saikosaponin C, CH54, 30.52%, 0.63), as the major active ingredients in the* Radix Bupleuri*, may be the main ingredients of the treatment of depression in the SNS formula. That may become the reason why* Radix Bupleuri* is the monarch drug in SNS formula.

Secondly, compounds from other herbal medicines may potentiate the* Radix Bupleuri* curing the main disease and other complications because it is possible to have the intersection of their pathogenesis. Many pharmacological researches have reported liquiritigenin (GC12, 67.9%, 0.18), naringenin (GC16, 53.00%, 0.21), kaempferol (SY12, 42.06%, 0.24), and formononetin (GC6, 68.33%, 0.21) have many clinical indications, such as antidepressant-like effect [25, 26] and attenuating Alzheimer's-like learning in memory deficits [27, 28]. In addition, paeoniflorin (SY26, 32.07%, 0.40) has significant sedative effect, hypnotic effect, and promoting nonrapid eye movement sleep effect as the function of regulating sleep disorder [29, 30]. Then, we found that Licochalcone A (GC8, 42.78%, 0.29), Licochalcone B (GC27, 75.13%, 0.26), neohesperidin (ZS8, 61.75, 0.27), and glycyrrhizic acid (GC32, 31.78%, 0.55) which are contained in* Radix Glycyrrhizae* and* Immature Bitter Orange* have the anti-inflammatory and antioxidant function. Neohesperidin also has the function of gastritis treatment [31–33]. Meanwhile, the mental disorders such as depression accompany the occurrence of inflammation [34]. Therefore, these ingredients from SNS formula play the important roles in the treatment of the complications of mental disorders.

Finally, 60 potential active ingredients (as shown in Table S1) from four herbal medicines mentioned above may become the core ingredients in treating mental disorders. It can show that the relieving effect of TCM can influence the symptoms and complications which can occur in the process of diseases. But it is still not clear that these different ingredients operate which kind of targets and how they adjust the bioprocess and organ function. Therefore, we use the potential active ingredients to predict the targets and analyze the different functions of these in order to explore the different functions of related organs.

### 3.2. Target Predictions and Locating the Main Organs

Undoubtedly, a multitarget from different organs binding by multi-ingredients from numerous herbal medicines is a noteworthy characteristic of TCM. And the holistic of TCM treatment mainly reflects on healing the diseases and disorders through regulating multifunctions on series organs [35]. But it is hard to clarify the holism treatment in molecule aspect by experimental methods. Thus, we have predicted the targets which were possibly influenced by the active ingredients above. 60 potential bioactive ingredients yielded 325 targets which were presented in Table S2. In order to investigate the potential role of herbal medicine in the human body, these targets were further standardized by UniProt blast tool and removed the targets which are not belonging to the human. Finally, we obtain 187 targets mainly expressed in the human body. The result is given in Table S3.

Further, for identifying the main expression tissue of those targets, we used the enrichment analysis by DAVID tools. As a result, we could identify 187 potential targets, and 116 of them are related to mental disorders and other complications which have similar symptoms in TCM. And through the enrichment analysis, there are 78 targets mainly expressed in brain, and 22 targets are mainly from the liver. 14 targets are from both brain and liver. In TCM theory, mental disorders are closely related to the liver and these indirectly reflected on the level of norepinephrine and epinephrine [36]. On the other hand, abnormal functions in the brain are also incentives and result in mental disorders [37]. Therefore, the emphasis of our study is these targets which are mainly expressed in the liver and brain.

### 3.3. Compound-Target Network Constructions and Analyses

In network topology analysis, the degree is a key topological parameter and represents the node number of the relations between one node and others in the network. In our work, degree value is used to identify the importance of potential targets and active ingredients. And the ingredients with higher degree mean that the more targets will be acted on it. Meanwhile, we consider that the target with a higher value of the degree means it would become the major potential target in the molecular treatment mechanism.

**Figure 1:**
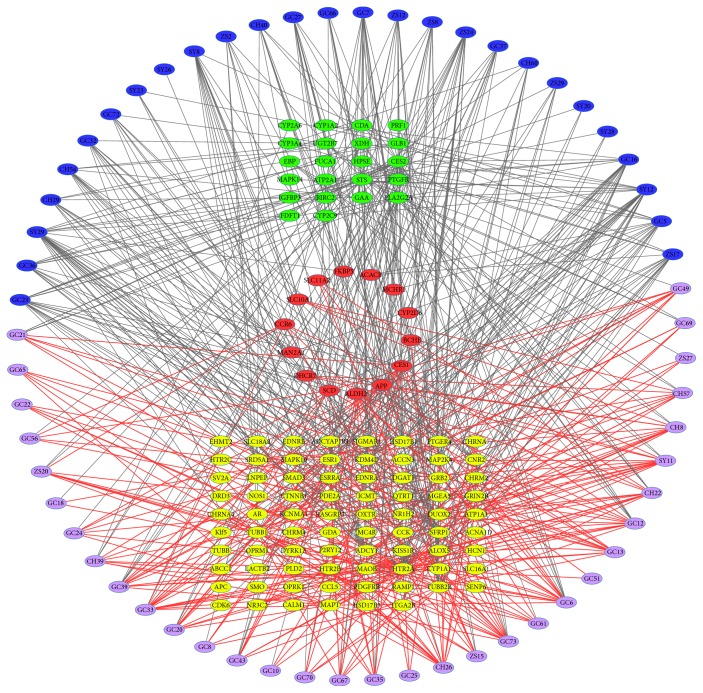
Compound-Target network of SNS formula. The blue and purple nodes represent the compounds from SNS formula. The purple nodes represent the compounds which can pass the BBB and the blue nodes with nonpenetrating compounds. The nodes colored with yellow (brain, node = 78), green (liver, node = 22), and red (both brain and liver, node = 14), respectively, represent the targets from different organs. The red line represents the interaction between BBB penetrating compounds and targets from the brain.

Further the targets and compounds based on the research above have been applied to construct the Compound-Target network (C-T network) (Figure 1), which contains 247 nodes (60 candidate active compounds and 187 potential targets) and 916 edges. As the result of DAVID enrichment analysis, the gene of these potential targets is mainly expressed in brain (92 targets) and liver (36 targets), and they account for 49.2% and 19.25% of the total number of targets. In other words, the active ingredient from SNS formula may prefer to act on the brain and liver.

In order to explain the holistic mechanism by four herbal medicines in using mental disorders and explore the multifunction of multitargets from numerous pathological changed organs, interestingly, according to the research by DAVID enrichment analysis, we found that the targets from brain and liver are the most of our research. And the number of the relationship between compounds and targets from liver and brain is 233 and 489, respectively, and their rates are 25.44% and 52.38%, respectively. Interestingly, hepatic encephalopathy is caused by the function abnormal of the liver and the brain and has the same symptoms as the mental disorder [38]. Therefore, it is not difficult to speculate that there must be some connections between mental disorders and they exist in the lesions of multiple organs in neurological disorders [39] and endocrine abnormal [40].

According to our literature research, targets from the brain with a high degree from the network have the bioactive in the process of mental disorders or related complications. First, 5-hydroxytryptamine receptor 2A (HTR2A) with the highest degree of 40 has been confirmed to participate in the process of major depression [41], attention deficit hyperactivity susceptibility [42], schizophrenia, mood disorders, anxiety disorders, eating disorders, suicide, and even Alzheimer's disease [43]. Tubulin beta-2B chain (TUBB2B) has the second highest degree of 26 in the C-T network targets. It has been reported to be mainly expressed in postmitotic neurons during neuronal migration and differentiation and then become a specific gene in brain development associated with epilepsy and neurodevelopmental delay [44]. They play an important role in the development of the nervous system and the transmission of neurotransmitters. Therefore, these targets may serve as therapeutic targets for these four herbal medicines above.

On the other hand, some potential indirect effects are also worth our attention. Estrogen receptor (ESR1) has the third highest degree of 20. Estrogen is mainly distributed in the brain and CNS with the function of direct regulation of the serotoninergic neurons, suppressing dopaminergic activity [45]. Thus, natural ingredients from SNS formula may play a role in an indirect effect of mental diseases treatment through the estrogen regulation. Meanwhile, the targets with a low degree also show the important clinical implications between patients with mental disorders and the normal group without mental disorders, such as neuronal acetylcholine receptor subunit alpha-4 (CHRNA4, degree = 3) and dopamine receptor D3 (DRD3, degree = 1) [46, 47]. Therefore, the lower degree targets may also play the indirect effect in the pathogenesis of mental disorders. On the other hand, the targets which are mainly expressed in the liver may have the functions in drug metabolism and other bioprocesses according to our text mining. And it may be the basis of traditional Chinese medicine adjuvant treatment of diseases [48, 49]. This indicates that our active ingredients may help in reducing side effects and toxicity of herbal medicine.

Interestingly, the degree of target from both brain and liver (mean degree = 6.79) is higher than the mean degree from single liver (mean degree = 6.27) or brain (mean degree = 5.05). That is to say, the natural ingredients are more likely binding with the common targets from both brain and liver. According to our literature research, SLC10A1 which is from both brain and liver is related to the affection neurobehavioral outcome due to the neuronal iron transportation adjustment, and it becomes an etiology factor of Parkinson's disease (PD) and learning mechanism of Alzheimer's disease (AD) [50, 51]. Therefore, it is worthwhile that we pay more attention to the targets from both brain and targets. In any case, the human body is an organic whole. It may be more effective than treating disease in a single aspect.

In the summary of C-T network, the holistic treatment of this formula is reflected in two parts. First and foremost, some ingredients binding with the targets from the brain in order to regular the nervous systems, most of them have BBB penetrating. And they may play the regulation of GABA interneuron followed by inhibition of NE neuronal activity according to HTR2A expression [43]. Meanwhile, some ingredients may be binding with TUBB2 and adjust the neuronal migration and differentiation to regularize the brain development and neurodevelopmental delay or have the function in mental disorders treatment. On the other hand, the ingredients with BBB nonpenetrating would rather bind to the targets from liver or other tissue and possibly have an adjustment function of monoamine and cholinergic neurotransmitters or drug metabolism promotion. Interestingly this phenomenon reflects the holistic regulation of multiple organs in Chinese medicine. Considering the accuracy of the prediction model, an animal model with chronic unpredictable mild stress (CUMS) will be used for treatment mechanism research and targets verification in our next investigation.

### 3.4. Related Diseases Prediction and Target-Disease Network

**Figure 2:**
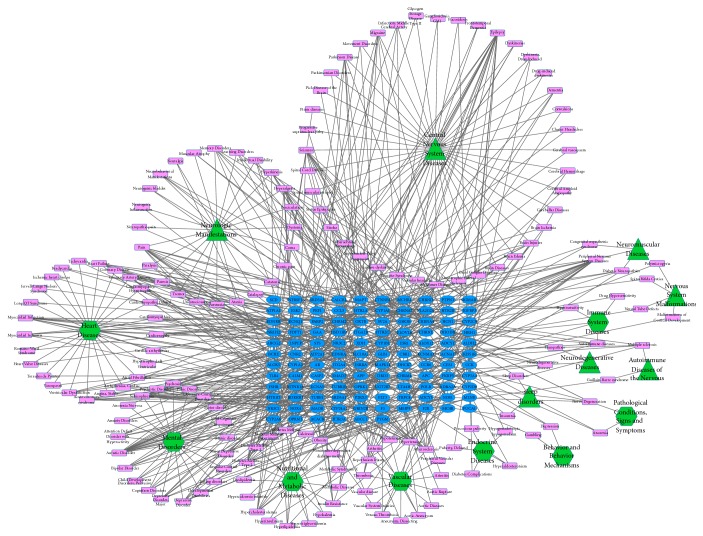
Target-Disease network of 116 potential targets and 170 related diseases which are classified into 15 groups by MeSH. Blue circle, purple rectangles, and green node represent the potential targets, related diseases, and the MeSH classification of diseases. And green triangles belong to nervous diseases.

**Figure 3:**
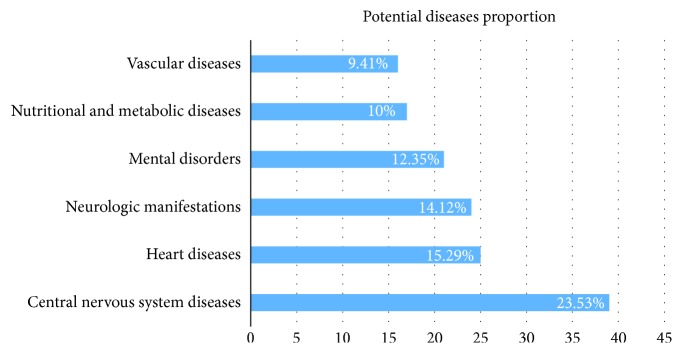
The statistical information of potential diseases from different six main MeSH classifications discovered by database.

Mental disorder is an aggregate of numerous related complex diseases, in which multigenes and their products in a state of dysfunction performed as an unbalance network [52]. Although mental disorders could be triggered by the compounds and targets mentioned above, there are still some potential diseases which are not included in recent clinical using, because herbal medicines have their own drug properties to regulate the specific unbalance status of the body and treat their main indications. In order to find potential disease in other mental disorders and related complications, we construct the Target-Disease network (T-D network) (Figure 2) in which the information is collected from TTD, CTD, and PharmGKB databases. In our study, 116 potential targets are related to 170 diseases. And the diseases are divided into 15 classes of MeSH. Among the 170 diseases, we found the top of classifications are central nervous system diseases (23.53%), neurologic manifestations (14.12%), mental disorders (12.35%), heart diseases (15.29%), nutritional and metabolic diseases (10%), and vascular diseases (9.41%). As shown in Figure 3, central nervous system diseases occupy the largest proportion. That is to say, our active natural ingredients from four herbal medicines can not only be used in the treating of mental disorders but also potentially treat other diseases with common targets.

In order to find the potential common mechanism of these diseases mentioned above. We constructed the T-D network combining targets and their related diseases. In this network, 14 diseases have more than 10 related targets (as shown in Table 1). And we found the potential targets were set intersection of these diseases and some of them are the key targets in mental disorders, such as CHRNA7 and HTR2A. The literature research has shown that the reduction of CHRNA7 expression possibly disinhibits the hippocampus through decreasing GAD-65 and GABAA receptor level and releasing activity-dependent GABA [53] (GABA is the major neurotransmitters in the mammalian central nervous system associated with major depression disorder [54]). As for the potential disease for SNS formula, autistic disorder and schizophrenia both have the highest degree value in the T-D network. And both of them belong to mental disorders. In addition, the abnormal of the nervous system is one of the main pathogeneses of mental disorder. So we consider SNS formula has the same regulation function in epilepsy, peripheral nervous system diseases, seizures, hyperalgesia, Alzheimer disease, and pain. We found that obesity and type 2 diabetes are reported as important complications among patients with mental illness [55] and long-term stressors on cardiac functioning become an important cause of cardiovascular disease [56]. And it becomes a major contributor to increasing mortality rate and decreased the life expectancy of 20% of mental disorder patients [57, 58]. It happens that stress in the environment can be expressed as an abnormal performance of multiple organs' functions and it reflected the abnormal function of the specific targets. Interestingly natural ingredients from four herbal medicines have the abilities to bind and adjust them. Then we analyzed the targets-diseases network by network topology method. The degree of diseases and their associated targets (gene symbol) was shown in Table 1.

In summary, common targets of these mental disorders are mostly binding by the ingredients from herbal medicines of SNS formula. Thus, SNS formula may relieve the common symptom of these mental disorders and treat them in common targets through same signal pathway. Interestingly, this may indirectly prove that these natural ingredients can be developed as the core drug of many kinds of mental diseases. In addition, SNS not only has the treatment of mental disorder, but also may have the potential treatment effect of metabolic diseases and cardiovascular diseases, such as type 2 mellitus, hypertension, heart failure, and myocardial infarction in other relevant systems. The result provides new information on clinical using for SNS formula and the prodrug discovery of its natural ingredients.

### 3.5. Molecular Mechanism for Herbal Medicines

At present, complex diseases are always accompanied with many kinds of symptoms and complications. Mental disorder is a collection of many complex diseases with similar symptoms to Qing-Zhi diseases in TCM. These four herbal medicines are the core ingredients from a classical TCM formula. It has been evolved that a variety of combinations formula for the treatment of mental disorders such as XiaoYaoSan and ChaiHuShuGanSan is used to treat depression [59, 60]. Meanwhile, four herbal medicines mentioned by our study are also the core of them. Therefore, we hope that we can find the active treatment ingredients of mental disorders through these herbal medicines.

Fortunately, systems pharmacology and a serial data analysis methods can help us to make a preliminary research on the herbal medicine ingredients, diagnosis, and TCM treatment mechanism of complex diseases. As we expected, we got 60 potential active ingredients from four herbal medicines with good pharmacokinetics parameters. And through our targets prediction and literature data mining, we found these compounds may have the connection of the targets with neurotransmitter transmission, neural development, neural iron transformation, and metabolism functions. As showed in Figure 1, there are many natural products which were combined with the same target as synergy action. Meanwhile, the degree can also reflect the number of natural ingredients which combined with one target. After network topology analysis, the targets from the brain have the mean degree of 5.31 and the targets from the liver with a mean degree of 6.47. It suggests that medical efficacy of herbal medicines is a variety of natural products combined with a large number of related targets. That is to say, TCM formula can achieve the purpose of treatment preferring multifunctions adjustment through different ways.

In order to discover and expand the application of natural drugs in the treatment of diseases, we found that four herbal medicines from SNS formula can not only regulate the nervous system as common clinical using but also have the effect in other related systems and organ, such as metabolic system, nervous system, vascular system, and heart. Interestingly, cerebrovascular disease, anxiety disorders, and mood disorder have been reported to be highly comorbid among adults [61]. Meanwhile, patients with mental illnesses also have increased the prevalence and risk factors of metabolic syndrome and its components [62]. That may reflect the characteristic of common drug targets for these diseases. Later we will analyze the differences of numerous modification formulas of four herbal medicines. It will correspond to the multigenes characteristic of precision medicine and treatment based on syndrome differentiation in TCM.

## 4. Conclusion

TCM formula is a multi-ingredient synergistic system that can play holistic treatment mechanism through regulating the multibiological process and function through multitarget from the different organ. The lacking of systematic research of TCM treatment limited the TCM using and drug discovering. Nevertheless, the systems pharmacology method can help us have a better understanding of TCM holism theory through three parts, potential active ingredients screening, potential targets prediction, and the molecule treatment mechanism exploring. The major finding of our recent work is summarized as follows.

1. After ADME screening, we got 60 potential active ingredients with favorable pharmacokinetics from four herbal medicines. As for the holism of this formula, the ingredients with BBB penetrating are more likely to operate in the brain and nervous system, and other ingredients with nonpenetrating ones focus more on liver regulation. This result may provide a method and basis of lead compound discovery in mental disorders.
2. The result of targets prediction shows that four herbal medicines probably act on 187 targets and most of them are from brain and liver. And the main functions of these targets are regulating neuronal activity through neurotransmitter, nervous protection, and influence of the neuronal migration and differentiation. That is to say, four herbal medicines may treat the mental disorders mainly through regulating the targets from brain and liver as a holism mechanism. In addition, because some targets play an important role in drug metabolism, these targets can also play a role in reducing the toxic and side effects and promoting the treatment effect.
3. In our potential diseases prediction, it is possible that four herbal medicines not only can be used for the treatment of mental disorders, but also have the potential treatment of cardiovascular diseases and nutritional metabolic diseases. There may be a link between these diseases, but they need to be further explored.
4. The holism theory and treatment based on differentiation are the most fundamental principle in TCM. Our research provides a systematic method in TCM formula study for mental disorders and series of complex diseases. This method may become an intermediary between TCM and modern medicine which may help us in promoting the understanding of TCM theory. However, our approach is limited to theoretical research, and therefore, we will use the classic pharmacological experiments to verify the results of the prediction in the next research.

## Supplementary Material

This table contains the ID, compound name, CAS number, source of ingredient, and relevant pharmacological parameters for the 60 ingredients. And the symbol “∗” from the first column is used to denote the corresponding hydrolyzate of the ingredient.

## Figures and Tables

**Table 1:** High correlation diseases with potential targets of SNS formula.

Disease name	Related targets (gene) symbol	MeSH class	Degree
Autistic disorder	APC, PRF1, HTR1D, CHRNA7, OXTR, DRD3, DHCR7, XDH, AR, CHRNA4, HTR3A, HTR2A, HTR2C, IGFBP3, KCNMA1, MAOB	Mental disorder	18
Schizophrenia	CHRNA7, CYP2D6, NOS1, BCHE, HRH1, HTR2A, HTR3A, NR3C1, OXTR, DRD3, GSK3B, MC4R, HTR2C, KCNMA1, MAOB, GRIN2B, CYP1A2	Mental disorder	18
Epilepsy	UGT2B7, GRIN2B, SV2A, CYP1A1, ABCG2, CHRM2, CYP2C9, CYP2A6, HRH1, OPRM1, ADRA2A, CHRNA4, BDKRB1, CHRNA7, CYP2D6, BCHE	Nervous system diseases	17
Peripheral nervous system diseases	ALOX12, PARP2, CYP1A2, SLC10A2, TLR4, CCL5, UGT2B7, TRPC4, ESR1, SRD5A2, SRD5A1, ELOVL6, CES1, ABCC1, CYP24A1, POLB	Nervous system diseases	17
Obesity	MLNR, MCHR1, CES1, STS, PTPN1, BCHE, HTR2C, ACACB, MC4R, MMP9, ESRRA, CCK, HTR2A, ESR1, KCNMA1	Nutritional and metabolic diseases	16
Pain	HRH1, BDKRB1, OPRK1, CHRNA4, CHRNA7, GRIN2B, F2R, OPRM1, CHRM4, P2RX3, CNR2, CHRM2	Nervous system diseases	13
Seizures	OPRM1, CHRNA7, CHRNA4, DRD3, CCK, CYP1A1, HCN1, NOS1, SIGMAR1, BCHE, OPRK1	Nervous system diseases	12
Atherosclerosis	TLR4, HRH1, F3, ESR1, ALOX5, NR1H2, CNR2, LTA4H, CES1, PLA2G2A, MMP9	Vascular diseases	12
Hypertension	HTR2B, NR3C1, HSD11B2, CYP3A4, CYP1A1, ADCY5, ABCC1, ADRA2A, AR, ATP1A1, EDNRA	Vascular diseases	12
Diabetes mellitus, type 2	PYGM, GAA, CYP2C9, TNFRSF1A, EDNRB, CYP1A2, PTPN1, EDNRA, KCNQ1, ADCY5	Nutritional and metabolic diseases	12
Hyperalgesia	ALOX12, P2RX3, OPRM1, CNR2, ALOX5, NOS1, DRD3, HTR2A, BDKRB1, GRIN2B	Nervous system diseases	11
Alzheimer disease	HTR2A, ESR1, GSK3B, BCHE, CYP2D6, CALM1, MAPT, CHRNA7, APP	Nervous system diseases	10
Heart failure	NR3C2, XDH, NOX4, GSK3B, ATP2A1, ADRA2A, ATP1A1, TNFRSF1A, HTR2B	Heart diseases	10
Myocardial infarction	ABCG2, GSK3B, CYP1A2, LTA4H, CYP2C9, NR3C2, HSD11B2, ESR1, MMP9	Heart diseases	10
